# Association between nocturnal sleep duration and the risk of hyperuricemia among Chinese government employees: A cross-sectional study

**DOI:** 10.3389/fpubh.2022.1055778

**Published:** 2022-11-23

**Authors:** Yanni An, Xuping Li, Feiyun Ouyang, Shuiyuan Xiao

**Affiliations:** Department of Social Medicine and Health Management, Xiangya School of Public Health, Central South University, Changsha, China

**Keywords:** nocturnal sleep duration, hyperuricemia, serum uric acid levels, association, government employees

## Abstract

**Objectives:**

Evidence has shown that nocturnal sleep duration is associated with the risk of hyperuricemia, yet the findings are inconsistent. Thus, we aimed at exploring the association between nocturnal sleep duration and the risk of hyperuricemia in Chinese government employees.

**Methods:**

A total of 10,321 government employees aged 20–60 years were collected from the Cohort Study on Chronic Diseases among Government Employees in Hunan Province, China. Sleep duration was self-reported. And serum uric acid levels >420 μmol/L in men and >360 μmol/L in women were considered hyperuricemia. The association between nocturnal sleep duration and hyperuricemia risk was examined utilizing multivariate logistic regression models. To further examine the connection between nocturnal sleep duration and serum uric acid levels, multiple linear regression analyses were utilized.

**Results:**

The prevalence of hyperuricemia was 17.2%. The results of logistic regression demonstrated that, in contrast to participants whose sleep duration was 7–8 h, those who slept for <7 h had an elevated risk of hyperuricemia (OR = 1.343, 95%CI: 1.126, 1.601). Further stratified analysis revealed that this association was still observed in those without obesity (OR = 1.365; 95%CI: 1.127, 1.655), hypertension (OR = 1.290, 95%CI: 1.054, 1.578), or diabetes mellitus (OR = 1.361, 95%CI: 1.136, 1.631). Multiple linear regression showed that shorter sleep duration (< 7 h) was positively correlated with serum uric acid levels. In comparison to individuals who slept for 7–8 h, those with sleep duration of fewer than 7 h had serum uric acid levels that were 7.231 μmol/L (95% CI: 2.875, 11.588) higher.

**Conclusion:**

Short nocturnal sleep duration (< 7 h) was associated with a higher risk of hyperuricemia, especially in participants without obesity, hypertension, or diabetes mellitus. Besides, short nocturnal sleep duration was related to greater uric acid levels.

## Introduction

Serum uric acid is the final metabolic product of purine (1). Hyperuricemia is a disease marked by unusually high serum uric acid levels (2), which can cause several diseases linked to crystal deposits, including uric acid nephropathy, gout, and urolithiasis (3). Many epidemiological studies have pointed out that hyperuricemia is intimately linked to the development of cardiovascular diseases (4), diabetes mellitus (DM) (5), hypertension (6), and dyslipidemia (7). It is one of the most common metabolic disorders in modern times with a heavy health and economic burden. The prevalence of hyperuricemia was 14.6% in the United States in 2015–2016 (8) and 16.6% in South Australia in 2008–2010 (9). In China, the prevalence of hyperuricemia was 11.1% in 2015–2016 and 14.0% in 2018–2019 (10), with an increasing trend. Therefore, preventing and managing hyperuricemia is an important public health issue.

The Health Ecology model (HEM) emphasizes that individual and population health is the outcome of the interaction among individual elements, material, and social environment. From the inside to the outside, this model has five levels: personal features, behavioral characteristics, interpersonal networks, living and working situations, and policy environment (11). Sleep, which is located in the second layer of the model, may have an impact on health. When we sleep, our body undergoes important metabolic regulation and hormone secretion, which play a vital role in maintaining a healthy balanced state (12). Both too little and too much sleep has been linked to obesity (13), hypertension (14), cardiovascular disease (13), DM (15), non-alcoholic liver disease (16), and dyslipidemia (17). Sleep deprivation activates proteolytic pathways, leading to an increase in byproducts of protein breakdown, such as purines, affecting the balance between uric acid synthesis, and degradation (18). Furthermore, sleep affects serum uric acid levels by regulating catecholamines, and cortisol levels (19). Therefore, we postulated that there might be a relationship between nocturnal sleep duration and hyperuricemia.

The relationship between sleep duration and hyperuricemia has been explored in various earlier research, but the findings remain contradictory. A study using the China Health and Nutrition Survey (CHNS) database found that individuals who slept for shorter periods of time had a higher risk of hyperuricemia (20). Results of the Kailuan cohort study revealed that the incidence of hyperuricemia was lower in long sleepers (21). In contrast, the correlation between sleep duration and hyperuricemia risk was insignificant in the Chinese Multi-Ethnic Cohort (CMEC) in the Yunnan region (22) and the National Health and Nutrition Examination Survey (NHANES) (23). A recent study reported that sleep duration was negatively correlated with serum uric acid levels (24), and another study with Korean women demonstrated a U-shaped association between sleep duration and serum uric acid levels (25). These associations may vary by race, gender, age, etc.

Government employees have the characteristics of long working h and lack of physical activity, so they are more likely to suffer from diseases than ordinary people (26, 27). Moreover, occupational staff have poorer sleep quality and shorter sleep duration (28). So far, there is no evidence on the association of nocturnal sleep duration with the risk of hyperuricemia and serum uric acid levels among Chinese government employees. In attempt to close this knowledge gap, we analyzed the baseline data of the Cohort Study on Chronic Diseases among Government Employees in Hunan Province, China, designed to look into any connections between nocturnal sleep duration, the risk of hyperuricemia, and serum uric acid levels among Chinese government employees.

## Materials and methods

### Study participants

The current study is based on data from the baseline survey of the Cohort Study on Chronic Diseases among Government Employees in Hunan Province, China, between 2018 and 2019. In China, government employees mainly refer to those who perform their public duties in a government department, state-owned company, or public institution in accordance with the law (such as civil servants, employees of public schools, or public hospitals) (29). Five cities in the Hunan Province of China (Changsha, Huaihua, Zhuzhou, Xiangtan, and Changde) were selected as the study sites. These cities were chosen based on the degree of participant collaboration, local infrastructures, and local economic development. Government employees from these cities were continuously invited to take part in our study from January 2018 to December 2019. We adopted the following inclusion criteria: (1) age 20–60 on the investigation day; (2) employees of the investigation institution; (3) without any cognitive impairment, being able to read and communicate normally; (4) those who completed the questionnaire and underwent physical examination; (5) those who voluntarily participated and signed the informed consent form. In five cities, a total of 30 departments were enlisted for the study. These departments included government agencies (860 employees), state-owned enterprises (1,818 employees), public institution (7,643 employees). 10,321 employees in total were recruited after filling out questionnaires. The work presented in this paper was approved by the Ethics Committee of the Xiangya School of Public Health, Central South University.

### Assessment of nocturnal sleep duration

Sleep duration was measured by using the following question “During the past month, when did you usually go to bed and wake up?” It was calculated as the time difference between waking up and going to bed normally. According to previous studies (30), sleep duration was divided into five groups: < 7, 7–8, 8–9, 9–10, and ≥10 h in our study. And sleep duration of 7–8 h was set as a reference. The use of hypnotics was divided into use and non-use over the past month. Moreover, this study used subjective sleep quality (including very good, fair, poor, and very bad) to evaluate participants' sleep quality.

### Definition of hyperuricemia

Following a minimum 12–hour overnight fast, blood samples were obtained from the antecubital vein of individuals for basic biochemical assays, which were drawn from 07:30 to 10:00 in the morning, and stored in a −20°C refrigerator until testing. Serum uric acid levels were measured by Hitachi 7,600–110 chemical autoanalyzer (Tokyo, Japan) with the use of enzyme colorimetry. Serum uric acid levels > 420 μmol/L for men and > 360 μmol/L for women were considered to have hyperuricemia (31).

### Covariates

The potential covariates in this study included participants' sociodemographic factors, occupational factors, lifestyle habits, mood symptoms, dietary habits, disease histories, and family histories, which were collected using the self-reported digital questionnaire.

Sociodemographic factors included gender, age, education level, marital status, and annual household income. The education level was divided into high school or below, university, and postgraduate or above. Marital status was categorized as unmarried, married/cohabitating, and divorced/widowed. Annual household income was classified into ≤100,000 RMB, 100,000–200,000 RMB, and >200,000 RMB.

Occupational factors included work intensity, daily sedentary time, and position levels. Work intensity was divided into brain work and physical work. The daily sedentary time was classified into four groups: < 2 h, 2–4 h, 4–6 h, and >6 h. Position levels were categorized into three levels: junior, middle, and senior or higher.

Several lifestyle habits were examined, including smoking, drinking, physical exercise participation, and napping. The participants' smoking habits were classified into current smokers and non-smokers; the latter included those who had smoked in the past and those who had never smoked. Participants who had smoked one or more cigarettes per day for the past year were considered current smokers. Drinking habits were categorized into two categories: current drinkers and non-drinkers (including those who had previously drunk and those who had never drunk). Those who consumed alcohol at least once per week in the past year were considered current drinkers, including beer, liquor, or other alcoholic beverages. Physical exercise was divided into two groups: participation and non-participation; those who exercised on average once a week or more over the previous year were considered physically active. The following questions were posed to participants to assess daytime napping: “Have you taken naps in the past six months?” If the answer is “Yes”, ask to report the average specific nap time, otherwise, mark it as 0.

Mood symptoms were represented by depression and anxiety conditions. The Patient Health Questionnaire-2 (PHQ-2) was utilized to determine whether participants had developed depressive symptoms within the previous 2 weeks. And the Generalized Anxiety Disorder-2 (GAD-2) was used to measure whether participants had experienced anxiety symptoms in the past 2 weeks. Participants were regarded as having mood symptoms if their PHQ-2 and/or GAD-2 scores met or exceeded 3, or they self-reported having depression or anxiety (32, 33).

The dietary habits included irregular meal, midnight snacks, and diet frequency. Regular meals were defined as three meals on time almost daily in the last 6 months. Eating late-night snacks more than once a week in the last 6 months was defined as eating midnight snacks. Diet frequency included weekly frequency of eating staple food, meat, poultry, seafood, eggs, dairy products, vegetables, beans, fruits, and desserts, which was divided into five categories: eating every day, 4–6days a week, 1–3 days a week, less than once a week/rarely/never.

Additionally, hypertension (Yes or No), obesity (Yes or No), dyslipidemia status (Yes or No), and DM (Yes or No) were objectively detected and evaluated in accordance with previous studies (34). Height and weight were measured using a high-definition liquid crystal intelligent body scale. Participants were asked to wear light clothes, and remove shoes and hats before measurement. This was measured twice to obtain an average value. Weight (kg) divided by height squared (m^2^) was used to establish the body mass index (BMI), and a BMI ≥ 28.0 kg/m^2^ was considered obesity (35). Hypertension was defined as either systolic blood pressure (SBP) ≥ 140 mmHg or diastolic blood pressure (DBP) ≥ 90 mmHg, or both self-reporting a diagnosis of hypertension, or taking medication for blood pressure (36). Fasting blood glucose (FPG) ≥ 7.0 mmol/L, self-reporting a diabetes diagnosis, or taking antidiabetic drugs were all considered to be indicators of DM (37). Total cholesterol (TC) levels ≥ 6.2 mmol/L, or triglyceride (TG) levels ≥ 2.3 mmol/L, or low-density lipoprotein cholesterol (LDL-C) levels ≥ 4.1 mmol/L, or high-density lipoprotein cholesterol (HDL-C) levels < 1.0 mmol/L, or a history of dyslipidemia, or taking anti- dyslipidemia medications were all considered to be dyslipidemia (38). Furthermore, family history was considered if either grandparents, parents, or siblings had hypertension, DM, obesity, hyperlipidemia (dyslipidemia), or gout.

### Statistical analyses

All analyses were conducted using IBM SPSS 25.0 and R 3.5.0. Covariate characteristics were expressed as mean ± standard deviation (M± SD) (the quantitative data) or proportion (%) (the qualitative data). To analyze the distinctions in characteristics across groups, student *t*-tests were employed for numerical variables and chi-square was utilized for categorical data. Multivariate logistic regression analyses were applied to assess the association between nocturnal sleep duration and the risk of hyperuricemia. Additionally, multiple linear regression analyses were utilized to look into the relationship between nocturnal sleep duration and serum uric acid levels. We constructed three models: model 1 was only adjusted for gender and age; model 2 was additionally adjusted by marital status, education level, annual household income, work intensity, sedentary time, position levels, participating in physical exercise, smoking, drinking, having mood symptoms, irregular meal habits, eating midnight snacks, using hypnotics, nap duration on the basis of model 1; model 3 was additionally adjusted for obesity, DM, hypertension, and dyslipidemia on the basis of model 2. Furthermore, to visually explore the dose-response relationship between nocturnal sleep duration and hyperuricemia risk, a restricted cubic spline (RCS) function was utilized, with five knots positioned at the 5%, 27.5%, 50%, 72.5%, and 95% percentiles of sleep duration (39). In order to assess the association in various individuals, studies were conducted across subgroups stratified by gender, age, obesity, dyslipidemia, hypertension, and DM, based on the findings of logistic regression analysis.

Two approaches were carried out in sensitivity analyses to assess the reliability of the findings. First, family history (including a family history of DM, hypertension, obesity, hyperlipidemia /dyslipidemia, and gout) and diet frequency, were further controlled in addition to the adjusted factors in the logistic regression models. Additionally, we corrected for potential confounders using the propensity score regression adjustment. In this method, propensity scores were computed using a logistic regression model with whether the nocturnal sleep duration was >7 h as the dichotomous dependent variable and the other covariates (gender, age, marital status, education level, annual household income, work intensity, sedentary time, position levels, participating in physical exercise, smoking, drinking, having mood symptoms, irregular meal habits, eating midnight snacks, using hypnotics, nap duration, obesity, hypertension, DM, and dyslipidemia) as the independent variable. A *P*-value <0.05 was regarded to be statistically significant.

## Results

### Characteristics of participants

A total of 10,321 people were included in the present study, of which 60.7% were women, the mean age (± SD) was 36.8 (± 9.6) years, the mean nocturnal sleep duration (± SD) was 7.70 (± 0.92) h and 17.2% had hyperuricemia (1,775/10,321). Table 1 indicates the study population's characteristics based on whether or not they had hyperuricemia. Compared with participants without hyperuricemia, those with hyperuricemia tended to be older and comprised a greater proportion of men, married, and brain work. Besides, participants with hyperuricemia were more likely to be sedentary, tended to smoke, and drink, had higher position levels, engaged in less physical exercise, had irregular eating habits, and used fewer hypnotics. They also had longer daytime naps, shorter nighttime sleep durations, and a greater prevalence of dyslipidemia, hypertension, obesity, and DM compared to participants without hyperuricemia (*P* < 0.05). The baseline characteristics of participants categorized by nocturnal sleep duration are shown in Supplementary Table 1.

**Table 1:** Characteristics of participants based on hyperuricemia.

**Characteristics**	**Non-hyperuricemia (*n* = 8,546)**	**Hyperuricemia (*n* = 1,775)**	***P*-value**
Age (year, mean ± SD)	36.60 ± 9.48	38.18 ± 10.01	<0.001
**Gender (** ***n*** **, %)**			<0.001
Men	2,811 (32.9)	1,240 (69.9)	
Women	5,735 (67.1)	535 (30.1)	
**Education level (** ***n*** **, %)**			0.193
High school or below	440 (5.1)	109 (6.1)	
University	5,467 (64.0)	1,110 (62.5)	
Postgraduate or above	2,639 (30.9)	556 (31.3)	
**Marital status (** ***n*** **, %)**			0.020
Unmarried	1,833 (21.4)	328 (18.5)	
Married/cohabitating	6,512 (76.2)	1,404 (79.1)	
Divorced/widowed	201 (2.4)	43 (2.4)	
**Annual household income (yuan**, ***n*****, %)**			0.287
≤100,000	3,609 (42.2)	761 (42.9)	
100,000–200,000	3,123 (36.5)	616 (34.7)	
>200,000	1,814 (21.2)	398 (22.4)	
**Work intensity (** ***n*** **, %)**			<0.001
Brain work	4,752 (55.6)	1,139 (64.2)	
Physical work	3,794 (44.4)	636 (35.8)	
**Sedentary time (** ***n*** **, %)**			0.012
<2 h	2,274 (26.6)	438 (24.7)	
2–4 h	3,880 (45.4)	773 (43.5)	
4–6 h	1,567 (18.3)	377 (21.2)	
>6 h	825 (9.7)	187 (10.5)	
**Position levels (** ***n*** **, %)**			<0.001
Junior	4,110 (48.1)	786 (44.3)	
Middle	2,967 (34.7)	620 (34.9)	
Senior or higher	1,469 (17.2)	369 (20.8)	
**Participating in**	4,002 (46.8)	886 (49.9)	0.018
**physical exercise (** ***n*** **, %)**			
**Current smoking (** ***n*** **, %)**	902 (10.6)	392 (22.1)	<0.001
**Current drinking (** ***n*** **, %)**	709 (8.3)	352 (19.8)	<0.001
**Having mood symptoms (** ***n*** **, %)**	516 (6.0)	99 (5.6)	0.456
**Irregular meal habits (** ***n*** **, %)**	3,839 (44.9)	739 (41.6)	0.011
**Midnight snacks (** ***n*** **, %)**	262 (3.1)	70 (3.9)	0.056
**Using hypnotics**	526 (6.2)	84 (4.7)	0.021
**Obesity (** ***n*** **, %)**	897 (10.5)	324 (18.3)	<0.001
**Hypertension (** ***n*** **, %)**	1,276 (14.9)	443 (25.0)	<0.001
**Diabetes mellitus (** ***n*** **, %)**	243 (2.8)	92 (5.2)	<0.001
**Dyslipidemia (** ***n*** **, %)**	1,518 (17.8)	823 (46.4)	<0.001
**Sleep quality (** ***n*** **, %)**			0.033
Very good	3,772 (44.1)	821 (46.3)	
Fair	3,788 (44.3)	773 (43.5)	
Poor	939 (11.0)	179 (10.1)	
Very bad	47 (0.5)	2 (0.1)	
**Sleep duration (h/night)**			<0.001
<7	913 (10.7)	247 (13.9)	
7–8	3,537 (41.4)	742 (41.8)	
8–9	3,054 (35.7)	608 (34.3)	
9–10	856 (10.0)	146 (8.2)	
≥10	186 (2.2)	32 (1.8)	
**Daytime napping (min, M** **±SD)**	28.58 ± 27.50	31.46 ± 28.14	<0.001

There were two types of data presented: mean (standard deviation) or number (percentage). In order to calculate a *P*-value, *t*-tests are used for continuous variables, and chi-square tests for categorical variables. M, mean; SD, standard deviation.

### Association between nocturnal sleep duration and hyperuricemia risk

**Figure 1:**
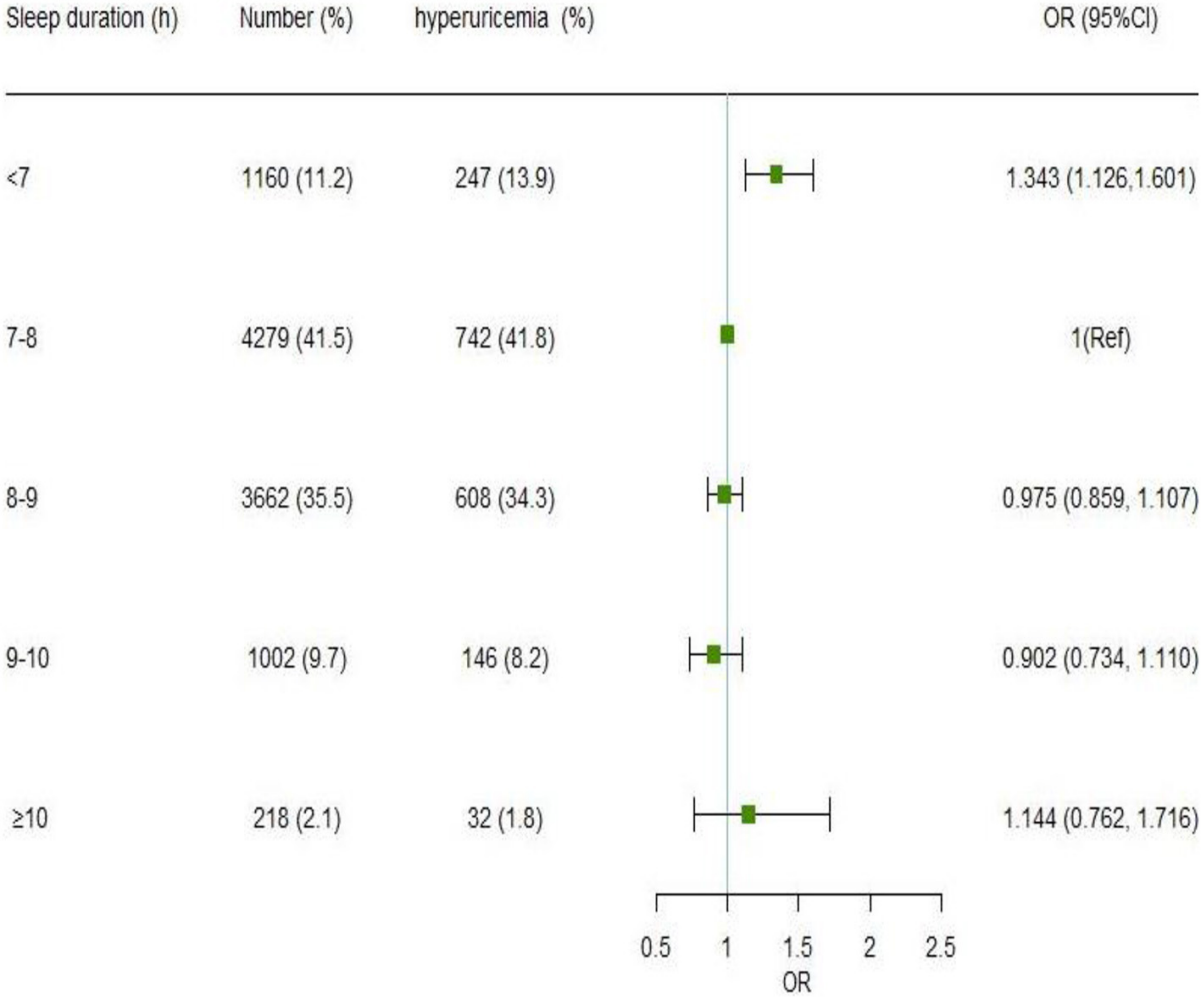
Association of nocturnal sleep duration with hyperuricemia by the logistic regression model. Model was adjusted for gender, age, marital status, education level, annual household income, work intensity, sedentary time, position levels, participating in physical exercise, smoking, drinking, having mood symptoms, irregular meal habits, midnight snacks, using hypnotics, nap duration, obesity, DM, hypertension, and dyslipidemia. The odds ratio point estimates are shown as small squares, while the 95% CIs are shown as horizontal lines. CI is for confidence interval; Ref stands for reference.

Figure 1 presents the association between nocturnal sleep duration and the risk of hyperuricemia. After adjusting for sociodemographic factors, occupational factors, lifestyle habits, and chronic diseases, participants who slept for <7 h had a 1.343 (95% CI: 1.126, 1.601) higher risk of hyperuricemia as compared to those whose sleep duration was 7–8 h. A higher risk of hyperuricemia was also seen in people with a sleep duration of ≥10 h (OR = 1.144, 95% CI: 0.762, 1.716), albeit this difference was not statistically significant. When fewer covariates were used, the findings remained similar (Supplementary Figure 1). Besides, the sensitivity analysis revealed that shorter sleep duration (< 7 h) was linked with hyperuricemia before (OR = 1.343, 95% CI: 1.126, 1.601) and after (OR = 1.317, 95% CI: 1.103, 1.573) additional adjustment for family history and dietary frequency. After adjusting confounding factors using the propensity score, the OR of hyperuricemia in the sleep duration of <7 h group was 1.318 (95% CI: 1.131, 1.537), compared to ≥7 h group (Supplementary Table 2).

**Figure 2:**
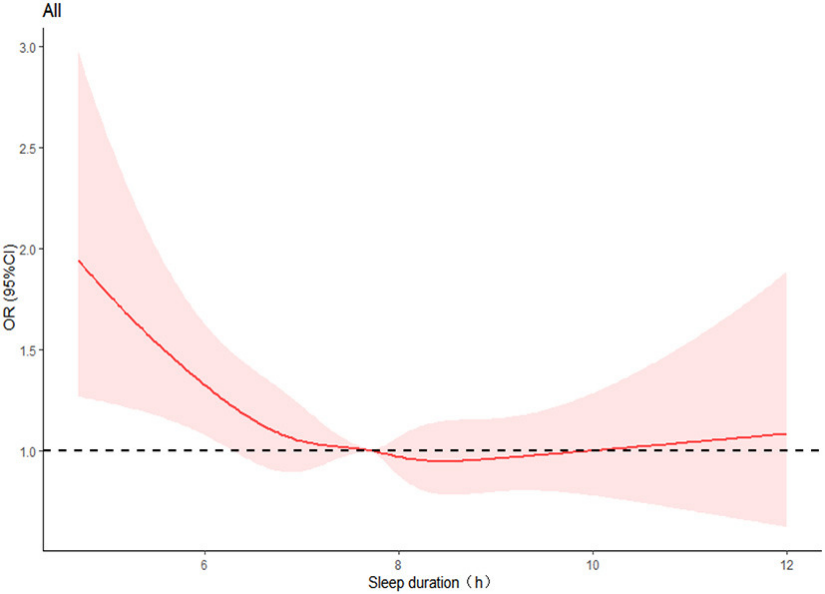
Restricted cubic spline plots of the relationship between nocturnal sleep duration and hyperuricemia. The curve was computed using restricted cubic spline (RCS) function that took into account variables including gender, age, marital status, education level, annual household income, work intensity, sedentary time, position levels, participating in physical exercise, smoking, drinking, having mood symptoms, irregular meal habits, midnight snacks, using hypnotics, nap duration, obesity, DM, hypertension, and dyslipidemia. The red area represents the 95% confidence interval for the odds ratio. The dotted line shows the level at which the OR value is equal to 1.

Figure 2 displays the dose-response relationship between nocturnal sleep duration and hyperuricemia risk after accounting for various factors. The restricted cubic spline indicated that the non-linearity was insignificant (*P* = 0.1207 > 0.05). But the curve has a tendency: with an increase of sleep duration, the OR value of hyperuricemia initially decreased and reached the lowest value when the sleep duration was about 8.5 h, and then appeared an upward tendency. The same trend was also observed in women (with a similar trough of around 8.5 h), but not in men (Supplementary Figure 2).

### Nocturnal sleep duration and hyperuricemia risk in subgroups

**Figure 3:**
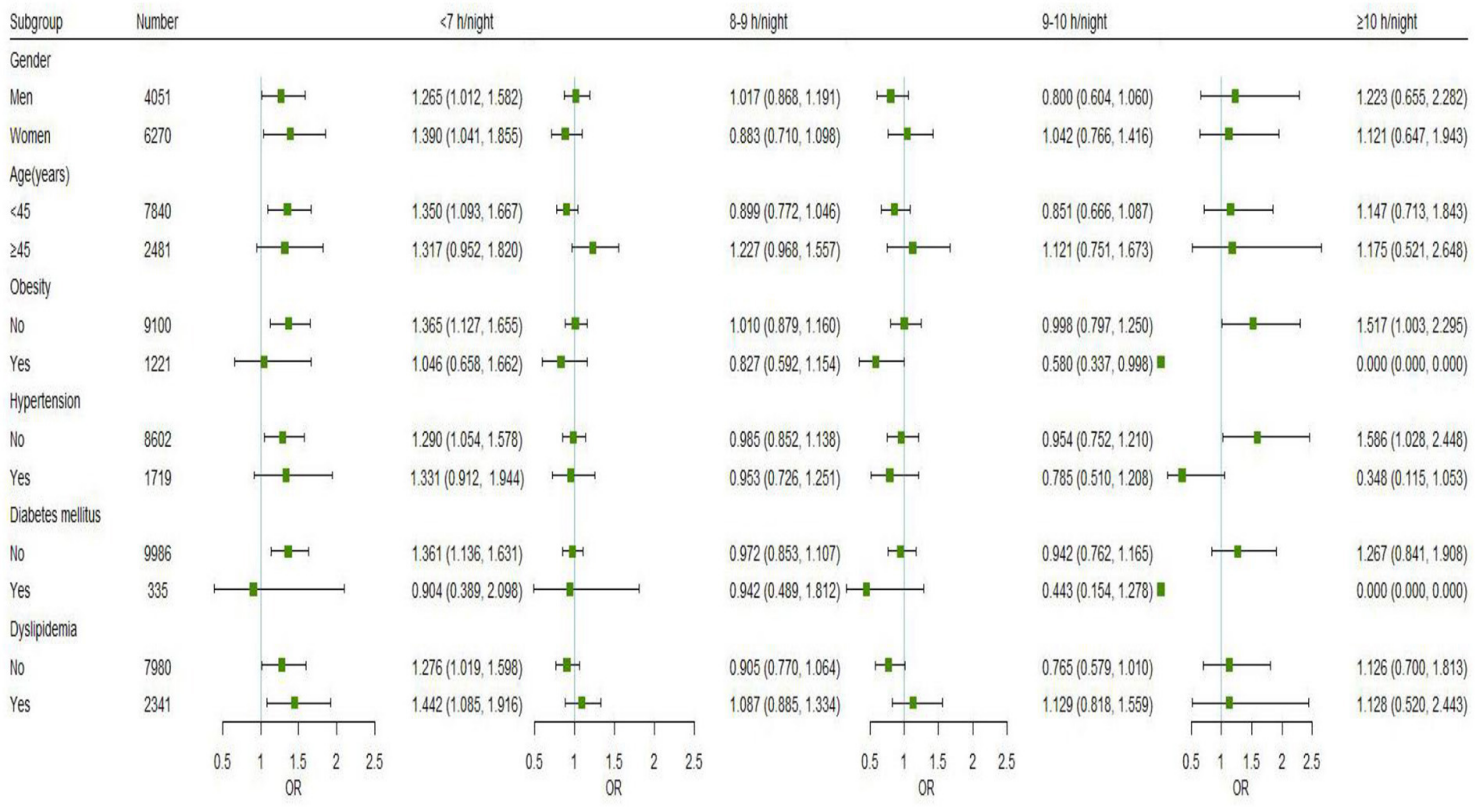
Subgroup analyses of the associations between nocturnal sleep duration and the risk of hyperuricemia were performed using logistic regression models based on gender, age, and chronic conditions (hypertension, dyslipidemia, DM, and obesity). All models were adjusted for marital status, education level, annual household income, work intensity, sedentary time, position levels, participating in physical exercise, smoking, drinking, having mood symptoms, irregular meal habits, midnight snacks, using hypnotics, nap duration, and were adjusted for gender, age, obesity, DM, hypertension, and dyslipidemia as appropriate. Point estimates for odds ratios are shown as small squares, while the 95% CIs are shown as horizontal lines. The small green squares represent sleep duration of <7 h/night, the blue represents sleep duration of 8–9 h/night, the red represents sleep duration of 9–10 h/night, and the black represents sleep duration of ≥10 h/night.

Figure 3 displays the findings for subgroups stratified by gender, age, and chronic conditions (hypertension, dyslipidemia, DM, and obesity) on the relationships between nocturnal sleep duration and hyperuricemia. Short nocturnal sleep duration (<7 h) was linked with a higher risk of hyperuricemia in men (OR = 1.265, 95% CI: 1.012, 1.582) and in women (OR = 1.390, 95% CI: 1.041, 1.855). Besides, the association was also observed in participants aged < 45 years (OR = 1.350, 95% CI: 1.093, 1.667), those without obesity (OR = 1.365, 95% CI: 1.127, 1.655), hypertension (OR = 1.290, 95% CI: 1.054, 1.578) and DM (OR = 1.361, 95% CI: 1.136, 1.631). Regardless of whether the individuals had dyslipidemia, it was discovered that short nocturnal sleep duration raised the risk of hyperuricemia, and the association seemed to be more pronounced in those with dyslipidemia (OR = 1.442, 95% CI: 1.085, 1.916). There was no interaction between nocturnal sleep duration and gender, age, hypertension, dyslipidemia, DM, or obesity (*P* > 0.05 for interaction).

### Relationship between nocturnal sleep duration and serum uric acid levels

The association between nocturnal sleep duration and serum uric acid levels was investigated using multiple linear regression analysis (Table 2). The results indicated that after adjusting for multiple variables, participants whose sleep duration was <7 h had 7.231 μmol/L higher serum uric acid levels (95% CI: 2.875, 11.588) compared to those with a sleep duration of 7–8 h. The results of the sensitivity analysis indicated after additional adjustment for family history and dietary frequency, higher uric acid levels were linked to short sleep duration (<7 h) (β = 6.707, 95% CI: 2.347, 11.067). After adjusting for confounding factors using the propensity score, the results did not change substantially (β = 8.343, 95% CI: 2.858, 13.828) (Supplementary Table 3).

**Table 2:** Association between nocturnal sleep duration and serum uric acid levels.

**Sleep duration (hours/night)**	**Number**	β **(95% CI)**		
		**Model 1**	**Model 2**	**Model 3**
<7	1,160 (11.2)	7.292 (2.823, 11.760)	7.541 (3.061, 12.022)	7.231 (2.875, 11.588)
7–8	4,279 (41.5)	0 (Ref)	0 (Ref)	0 (Ref)
8–9	3,662 (35.5)	−2.194 (−5.233, 0.845)	−2.047 (−5.098, 1.005)	−2.387 (−5.354, 0.580)
9–10	1,002 (9.7)	−0.796 (−5.540, 3.949)	−0.195 (−4.958, 4.568)	−1.914 (−6.547, 2.720)
≥10	218 (2.1)	1.417 (−7.967, 10.801)	1.897 (−7.484, 11.278)	1.682 (−7.439, 10.802)

Model 1 was only adjusted for gender and age; Model 2 was additionally adjusted by marital status, education level, annual household income, work intensity, sedentary time, position levels, participating in physical exercise, smoking, drinking, having mood symptoms, irregular meal habits, midnight snacks, using hypnotics, nap duration on the basis of Model 1; Model 3 was additionally adjusted for obesity, DM, hypertension, and dyslipidemia on the basis of Model 2.

## Discussion

The findings of this study demonstrated that insufficient sleep duration (< 7 h) was linked to a higher risk of hyperuricemia, and raised serum uric acid levels. Additionally, subgroup analysis revealed that a significant association between short nocturnal sleep duration and hyperuricemia still existed among participants without obesity, hypertension, or DM, which offers knowledge about protecting vulnerable people and screening high-risk populations. In all, our research implied that proper sleep extension might be a possible public health measure to lower the risk of hyperuricemia.

Adequate sleep can eliminate fatigue and improve body immunity, which is beneficial to health. Previous studies have proven that short sleep duration was linked to a variety of chronic conditions (17, 37, 40). Li et al. (36) and Fernandez-Mendoza et al. (41) demonstrated that hypertension risk increased when people slept for shorter periods. Liu et al. (37) and Yadav et al. (42) reported that diabetes risk increased with shorter sleep duration. Besides, inadequate sleep duration is linked to metabolic syndrome (40) and obesity (43). Previous research has demonstrated a correlation between nocturnal sleep duration and hyperuricemia risk. The main outcome of this study was in line with Yu et al.'s study of 8,289 adults aged ≥18 years, in which shorter sleep duration was linked to a higher risk of hyperuricemia (20). A study of 6,151 Korean women older than 20 years (25) revealed that long night sleep duration elevated the incidence of hyperuricemia (OR = 1.94, 95%CI: 1.27, 2.96), but our research found the relationship was insignificant. Dong et al. (44) found there was no correlation between nocturnal sleep duration and hyperuricemia risk in a survey involving 29,643 adults aged 18–79 in rural Henan province, which was congruent with the results of a study conducted by Wang et al. in a multi-ethnic population of 22,038 people aged 30–79 years (22). Additionally, the current study discovered that short nocturnal sleep duration was linked to increased serum uric acid levels, which was in agreement with the results on 4,555 Taiwanese people aged ≥18 years (45). In the study of 1,842 older people with high cardiovascular risk, Papandreou et al. (24) observed an inverse relationship between nocturnal sleep duration and serum uric acid levels. The reason for the inconsistent outcomes of this research may be related to the selection of participants, the grouping of sleep duration, and the confounding variables controlled for. In addition, more investigations with large sample populations are required to identify the association between long sleep duration and hyperuricemia risk.

The mechanisms of short sleep duration and hyperuricemia remain unclear. There are, however, several possible conjectures. Sleep deprivation is associated with elevated catecholamine levels, which help break down nucleotides, thus promoting the generation of endogenous uric acid (19). Catecholamines have also been shown to be crucial developing hyperuricemia in animal models (46). Additionally, lack of sleep can activate proteolytic pathways, synthesizing of uric acid, and purines (18). The relationship between sleep duration and serum uric acid levels could also be mediated by inflammation. Studies have reported that sleep duration can significantly impact on inflammatory mediators, thereby resulting in various chronic inflammatory illnesses (47, 48). Uric acid participates in the body's inflammatory response as an immune system activator (49), increasing uric acid levels in the serum. Furthermore, insufficient sleep duration may decrease levels of melatonin and leptin as well as elevate levels of ghrelin and cortisol, which may further result in obesity, diabetes, hyperinsulinemia, dyslipidemia, and hypertension (50), thereby aggravating the burden on the kidney.

Results of subgroup analyses revealed that short nocturnal sleep duration (< 7 h) had a higher risk of hyperuricemia among participants aged <45 years. The association seemed to be more pronounced in women and people with dyslipidemia. Additionally, such association was also found among individuals without hypertension, obesity, or DM. Similar to our results, Liu et al. (51) revealed that insufficient sleep duration was connected with an increased risk of hyperuricemia in young people (< 45 years old) among community residents. Previous research that explored the relationship between sleep duration and hyperuricemia also produced inconsistent findings between men and women (20, 51). Men and women have varied sex hormone levels and types, and their bodies react differently to them (52). These factors may lead to gender variations in serum urate excretion. We discovered that those with dyslipidemia had a greater risk of hyperuricemia when they slept for short periods of time, which was lined with earlier research (7). Dyslipidemia can lead to lipid accumulation, which reduces urate excretion by the kidneys and raises serum uric acid levels (53). Yu et al. (20) revealed that hyperuricemia risk is increased by insufficient sleep time among participants without DM, obesity, and hypertension, suggesting that individuals without traditional risk factors are still at higher risk of hyperuricemia when they sleep less. Although the exact mechanisms are indistinct, it is plausible that the presence of risk factors for chronic diseases or lifestyle changes could obscure the impact of sleep duration in high-risk individuals while leaving a discernible impact in relatively healthy individuals. In addition, sleep duration could be an independent risk factor for hyperuricemia irrespective of these chronic disease factors.

This study added to the evidence of the association between nocturnal sleep duration, the risk of hyperuricemia, and serum uric acid levels in government employees. In addition, our study had a relatively large sample, and many covariates were controlled in the analysis. However, there are still some limitations. First of all, because this study was cross-sectional in nature, it cannot adequately depict the causal association of hyperuricemia and serum uric acid levels with sleep duration. Secondly, nocturnal sleep duration was obtained through self-reporting, without using objective medical equipment to measure, such as polysomnography monitor, or sleep tester; thus, there may exist some recall bias. Thirdly, the study was conducted on a special group of government employees, so care should be taken when inferring these findings to other groups.

## Conclusions

In a word, this study demonstrated that short nocturnal sleep duration was associated with an elevated risk of hyperuricemia, and increased serum uric acid levels in Chinese government employees. The association between nocturnal sleep duration and hyperuricemia remained in participants without hypertension, obesity, or DM. Further confirmation of the longitudinal relationship between nocturnal sleep duration and hyperuricemia risk in this population is required.

## Data availability statement

The raw data supporting the conclusions of this article will be made available by the authors, without undue reservation.

## Ethics statement

The studies involving human participants were reviewed and approved by the Ethics Committee of the Xiangya School of Public Health, Central South University. The patients/participants provided their written informed consent to participate in this study.

## Author contributions

YA was responsible for data analyses, manuscript writing, and revision. FO and XL were responsible for the manuscript revision. SX was responsible for the study conceptualization and manuscript revision. All authors participated in the data collection. All authors read and approved the final manuscript.

## Funding

This research was funded by the Ministry of Science and Technology of China (Grant No. 2016YFC0900802). The funders had no role in the design of the study, data collection and analysis, decision to publish, and in writing the manuscript.

## Conflict of interest

The authors declare that the research was conducted in the absence of any commercial or financial relationships that could be construed as a potential conflict of interest.

## Publisher's note

All claims expressed in this article are solely those of the authors and do not necessarily represent those of their affiliated organizations, or those of the publisher, the editors and the reviewers. Any product that may be evaluated in this article, or claim that may be made by its manufacturer, is not guaranteed or endorsed by the publisher.
