# Supplementary material for: Association between nocturnal sleep duration and the risk of hyperuricemia among Chinese government employees: A cross-sectional study
Source: Front Public Health. 2022 Nov 23;10:1055778. doi: 10.3389/fpubh.2022.1055778 (PMC9727396; doi:10.3389/fpubh.2022.1055778)
Supplement: Supplementary file 1 [file Data_Sheet_1.PDF]

Supplementary Material

Table 1 Baseline characteristics of participants categorized by nocturnal sleep duration.

**Characteristics**	**Nocturnal sleep duration (hours)**					**P**
	**< 7**	**7 – 8**	**8 – 9**	**9 – 10**	**≥ 10**	
Age (year, mean±SD)	37.48±9.72	36.87±9.54	37.05±9.61	35.98±9.60	35.00±9.50	<0.001
Gender (n, %)						<0.001
Men	475 (40.9)	1772 (41.4)	1433 (39.1)	318 (31.7)	53 (24.3)	
Women	685 (59.1)	2507 (58.6)	2229 (60.9)	684 (68.3)	165 (75.7)	
Education level (n, %)						<0.001
High school or below	63 (5.4)	170 (4.0)	211 (5.8)	78 (7.8)	27 (12.4)	
University	738 (63.6)	2681 (62.7)	2345 (64.0)	658 (65.7)	155 (71.1)	
Postgraduate or above	359 (30.9)	1428 (33.4)	1106 (30.2)	266 (26.5)	36 (16.5)	
Marital status (n, %)						<0.001
Unmarried	277 (23.9)	999 (23.3)	664 (18.1)	178 (17.8)	43 (19.7)	
Married/cohabitating	845 (72.8)	3186 (74.5)	2907 (79.4)	807 (80.5)	171 (78.4)	
Divorced/widowed	38 (3.3)	94 (2.2)	91 (2.5)	17 (1.7)	4 (1.8)	
Annual household income (yuan, n, %)						<0.001
≤100,000	484 (41.7)	1702 (39.8)	1608 (43.9)	463 (46.2)	113 (51.8)	
100,000-200,000	439 (37.8)	1600 (37.4)	1279 (34.9)	353 (35.2)	68 (31.2)	
>200,000	237 (20.4)	977 (22.8)	775 (21.2)	186 (18.6)	37 (17.0)	
Work intensity (n, %)						<0.001

**Characteristics**	**Nocturnal sleep duration (hours)**					**P**
	**< 7**	**7 – 8**	**8 – 9**	**9 – 10**	**≥ 10**	
Brain work	651 (56.1)	2604 (60.9)	2080 (56.8)	473 (47.2)	83 (38.1)	
Physical work	509 (43.9)	1675 (39.1)	1582 (43.2)	529 (52.8)	135 (61.9)	
Sedentary time (n, %)						<0.001
< 2 h	231 (19.9)	1026 (24.0)	1090 (29.8)	302 (30.1)	63 (28.9)	
2–4 h	505 (43.5)	1991 (46.5)	1617 (44.2)	443 (44.2)	97 (44.5)	
4–6 h	261 (22.5)	822 (19.2)	638 (17.4)	185 (18.5)	38 (17.4)	
> 6 h	163 (14.1)	440 (10.3)	317 (8.7)	72 (7.2)	20 (9.2)	
Position levels (n, %)						<0.001
Junior	558 (48.1)	1976 (46.2)	1684 (46.0)	545 (54.4)	133 (61.0)	
Middle	399 (34.4)	1454 (34.0)	1337 (36.5)	333 (33.2)	64 (29.4)	
Senior or higher	203 (17.5)	849 (19.8)	641 (17.5)	124 (12.4)	21 (9.6)	
Participating physical exercise (n, %)	469 (40.4)	2096 (49.0)	1773 (48.4)	456 (45.5)	94 (43.1)	<0.001
Current smoking (n, %)	204 (17.6)	563 (13.2)	408 (11.1)	101 (10.1)	18 (8.3)	<0.001
Current drinking (n, %)	146 (12.6)	456 (10.7)	359 (9.8)	83 (8.3)	17 (7.8)	0.007
Having mood symptoms (n, %)	119 (10.3)	246 (5.7)	192 (5.2)	47 (4.7)	11 (5.0)	<0.001
Irregular meal habits (n, %)	633 (54.6)	1996 (46.6)	1427 (39.0)	411 (41.0)	111 (50.9)	<0.001
Midnight snacks (n, %)	77 (6.6)	164 (3.8)	69 (1.9)	17 (1.7)	5 (2.3)	<0.001
Using hypnotics	101 (8.7)	264 (6.2)	179 (4.9)	53 (5.3)	13 (6.0)	<0.001

Characteristics	Nocturnal sleep duration (hours)					P
	**< 7**	**7 – 8**	**8 – 9**	**9 – 10**	**≥ 10**	
Obesity (n, %)	134 (11.6)	528 (12.3)	399 (10.9)	130 (13.0)	30 (13.8)	0.181
Hypertension (n, %)	186 (16.0)	714 (16.7)	601 (16.4)	177 (17.7)	41 (18.8)	0.746
Diabetes mellitus (n, %)	48 (4.1)	120 (2.8)	119 (3.2)	39 (3.9)	9 (4.1)	0.113
Dyslipidemia (n, %)	290 (25.0)	944 (22.1)	834 (22.8)	236 (23.6)	37 (17.0)	0.061
Hyperuricemia (n, %)	247 (21.3)	742 (17.3)	608 (16.6)	146 (14.6)	32 (14.7)	<0.001
Sleep quality (n, %)						<0.001
Very good	322 (27.8)	1821 (42.6)	1773 (48.4)	549 (54.8)	128 (71)	
Fair	557 (48.0)	1976 (46.2)	1586 (43.3)	371 (37.0)	71 (32.6)	
Poor	266 (22.9)	466 (10.9)	292 (8.0)	76 (7.6)	18 (8.3)	
Very bad	15 (1.3)	16 (0.4)	11 (0.3)	6 (0.6)	1 (0.5)	
Daytime napping (min, M±SD)	28.92±27.66	29.53±26.65	29.33±27.97	26.46±29.33	28.67±31.77	0.032

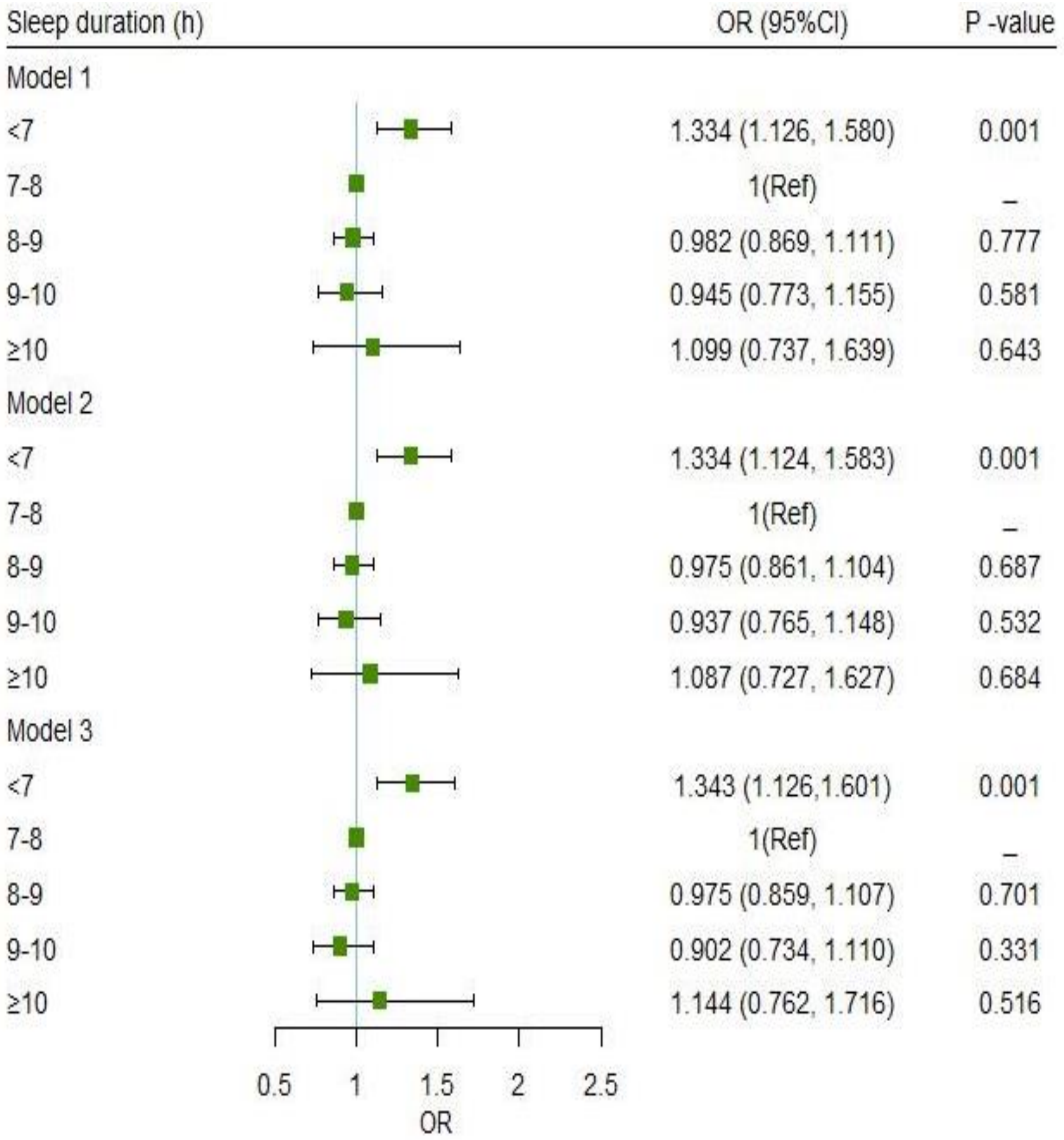

Figure 1. The association between nocturnal sleep duration and the risk of hyperuricemia is represented by three logistic regression models. Model 1 was only adjusted for gender and age; Model 2 was additionally adjusted by marital status, education level, annual household income, work intensity, sedentary time, position levels, participating in physical exercise, smoking, drinking, having mood symptoms, irregular meal habits, midnight snacks, using hypnotics, nap duration on the basis of Model 1; Model 3 was additionally adjusted for obesity, DM, hypertension, and dyslipidemia on the basis of Model 2. The odds ratio point estimates are shown as small squares, while the 95% CIs are shown as horizontal lines. h, hours; OR, odds ratio; CI, confidence interval; Ref, reference.

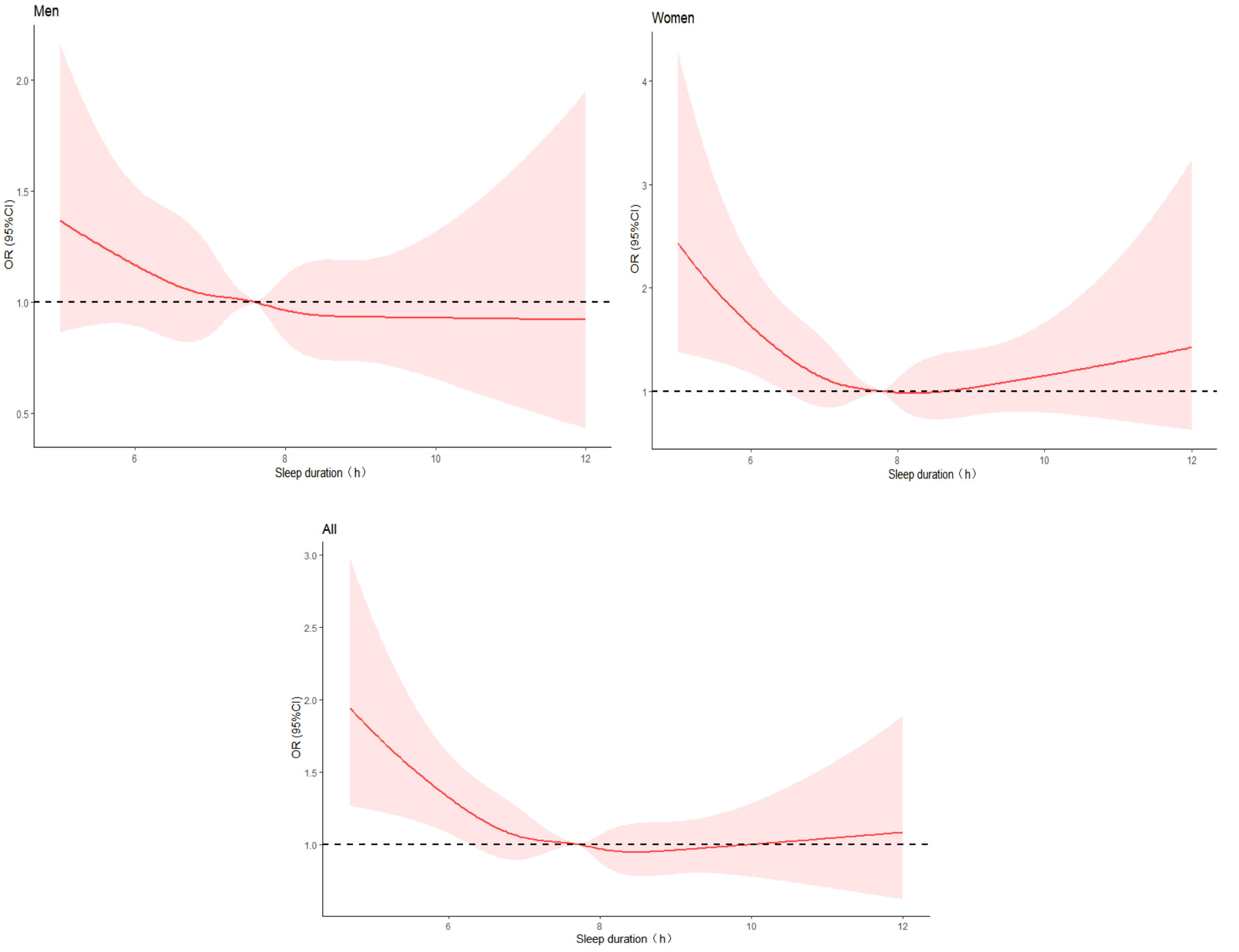

Figure 2 The dose-response relationship between nocturnal sleep duration and hyperuricemia by gender. The curve was computed using a restricted cubic spline (RCS) function that took into account variables including gender, age, marital status, education level, annual household income, work intensity, sedentary time, position levels, participating in physical exercise, smoking, drinking, having mood symptoms, irregular meal habits, midnight snacks, using hypnotics, nap duration, obesity, DM, hypertension, and dyslipidemia. The red area represents the 95% confidence interval for the odds ratio. The dotted line shows the level at which the OR value is equal to 1.

Table 2 Sensitivity analysis of the association between nocturnal sleep duration and the risk of hyperuricemia

Sleep duration (hours)	OR	95% CI of OR	P-value
**Further adjusted for family history and diet frequency***			
< 7	1.317	1.103–1.573	0.002
7– 8	1 (Ref)	1 (Ref)	1 (Ref)
8– 9	0.978	0.860–1.112	0.734
9– 10	0.910	0.738–1.122	0.378
≥ 10	1.127	0.747–1.702	0.568
**Propensity score regression adjustment****			
< 7	1.318	1.131–1.537	<0.001
≥ 7	1 (Ref)	1 (Ref)	1 (Ref)

* Multivariable logistic regression model was adjusted for gender, age, marital status, education level, annual household income, work intensity, sedentary time, position levels, participating in physical exercise, smoking, drinking, having mood symptoms, irregular meal habits, midnight snacks, using hypnotics, nap duration, obesity, DM, hypertension, and dyslipidemia, and further adjusted for family history (including family history of obesity, DM, hypertension, hyperlipemia(dyslipidemia) and gout) and diet frequency factors (including weekly frequency of eating staple food, meat, poultry, seafood, eggs, dairy products, vegetables, beans, fruits, and desserts).

** The propensity scores were computed using a logistic regression model with the dichotomous dependent variable of whether the sleep duration was greater than 7 hours.

Table 3 Sensitivity analysis of the association between nocturnal sleep duration and serum uric acid levels

Sleep duration (hours)	β	95% CI of β	P-value
**Further adjusted for family history and diet frequency***			
< 7	6.707	2.347–11.067	0.003
7 – 8	0 (Ref)	0 (Ref)	0 (Ref)
8 – 9	-2.095	-5.066–0.877	0.167
9 –10	-1.418	-6.069–3.233	0.550
≥ 10	2.520	-6.611–11.652	0.589
**Propensity score regression adjustment** **			
< 7	8.343	2.858–13.828	0.003
≥ 7	0 (Ref)	0 (Ref)	0 (Ref)

* Multivariable logistic regression model was adjusted for gender, age, marital status, education level, annual household income, work intensity, sedentary time, position levels, participating in physical exercise, smoking, drinking, having mood symptoms, irregular meal habits, midnight snacks, using hypnotics, nap duration, obesity, DM, hypertension, and dyslipidemia, and further adjusted for family history (including family history of obesity, DM, hypertension, hyperlipemia(dyslipidemia) and gout) and diet frequency factors (including weekly frequency of eating staple food, meat, poultry, seafood, eggs, dairy products, vegetables, beans, fruits, and desserts).

** The propensity scores were computed using a logistic regression model with the dichotomous dependent variable of whether the sleep duration was greater than 7 hours.
